# Prehabilitation in Implant Dentistry: An Essential Strategy for Primordial Prevention of Peri‐Implant Diseases

**DOI:** 10.1111/jre.70048

**Published:** 2025-10-17

**Authors:** Maria Clotilde Carra, Philippe Bouchard

**Affiliations:** ^1^ Departement of Translational Medicine University of Ferrara Ferrara Italy; ^2^ METHODS Team, CRESS Université Paris Cité Paris France; ^3^ Université Paris Cité, Inserm UMR 1333 Santé Orale Paris France

## Abstract

Peri‐implant diseases (PIDs) are highly prevalent and threaten both the success and longevity of implant‐supported prostheses. Their prevention should begin before implant surgery (i.e., primordial prevention) by avoiding risk factor exposure and ensuring optimal implant placement conditions. Prehabilitation, a multimodal strategy already used in other surgical fields, can be applied to implant dentistry to optimize patient status before surgery. By addressing modifiable behavioral risk factors and strengthening systemic and local conditions, prehabilitation would enhance both short‐ and long‐term outcomes of implant‐supported rehabilitation.

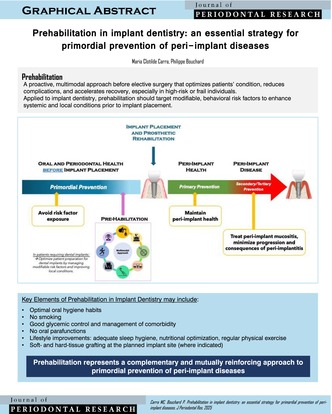

In 2023, the European Federation of Periodontology (EFP) published the S3‐level clinical practice guideline for the prevention and treatment of peri‐implant diseases (PIDs) [1]. In this document, the concept of primordial prevention was introduced for the first time in implant dentistry [2], emphasizing that prevention of PIDs should begin prior to implant placement to avoid risk factor exposure. Indeed, the prevention framework of PIDs is structured into four levels: (i) primordial prevention, which takes place before implant placement and aims to prevent the development of risk factors; (ii) primary prevention, which involves measures applied after implant placement to ensure that healthy, functional implants do not develop disease (e.g., through supportive peri‐implant care, SPIC); (iii) secondary prevention, which implies the management of early disease (e.g., peri‐implant mucositis) to prevent disease progression and recurrence; and (iv) tertiary prevention, which focuses on the consequences of an established disease (e.g., peri‐implantitis) with the aim of minimizing the sequelae (Figure 1).

**FIGURE 1:**
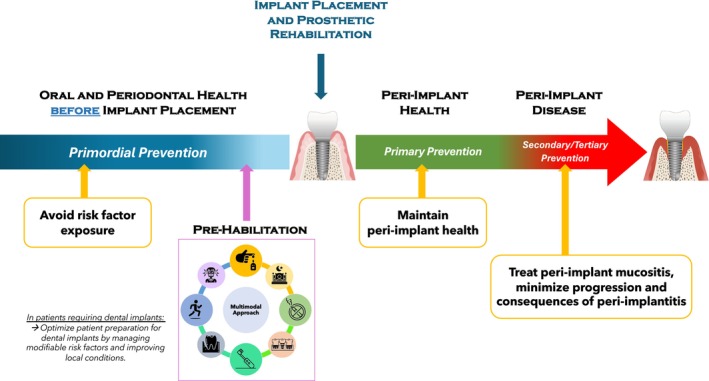
Schematic representation of prevention strategies in implant dentistry and the implementation of prehabilitation. Pre‐habilitation is a multimodal approach implemented prior to implant placement, aimed at optimizing the preparation of patients who are candidates for implant‐supported rehabilitation. This approach encompasses both the control of systemic and behavioral risk factors as well as the enhancement of local conditions. Key elements may include: Establishing optimal oral hygiene habits, smoking cessation, glycemic control, management of oral parafunctions (e.g., bruxism), promotion of adequate sleep hygiene, nutritional optimization, regular physical exercise, and, where indicated, soft‐ and hard‐tissue grafting at the planned implant site. Prehabilitation represents a complementary and mutually reinforcing approach to primordial prevention of PIDs, both aiming to avoid risk factor exposure prior to implant placement and thereby minimize the occurrence of PIDs over time. Once the dental implant is placed and functional, primary prevention focuses on controlling risk factors to maintain peri‐implant health and prevent disease. After disease onset, early detection and treatment (e.g., of peri‐implant mucositis) or prevention of recurrence constitute secondary prevention. Tertiary prevention, in turn, aims to minimize the consequences of disease (i.e., peri‐implantitis) and rehabilitate any resulting damage.

Primordial prevention is achieved through self‐directed lifestyle choices that avert the development of risk factors. Traditionally, it is conceived as a population‐level strategy to prevent the onset of disease. This approach has been explored in several areas of medicine, especially in the context of non‐communicable chronic diseases, such as cardiovascular diseases. It is particularly relevant for oral diseases, which are highly prevalent, associated with multiple behavioral and environmental risk factors, and carry a considerable clinical and economic burden for patients and healthcare systems [3, 4]. However, it has been poorly investigated and is not yet systematically applied in research or clinical practice in implant dentistry.

PIDs encompass peri‐implant mucositis and peri‐implantitis, both of which are inflammatory conditions affecting the tissues surrounding dental implants. While peri‐implant mucositis is confined to the soft tissues, peri‐implantitis is characterized by progressive bone loss that ultimately jeopardizes implant survival. Alarmingly, the prevalence of PIDs is high. It is estimated that approximately 50% of patients with dental implants experience peri‐implant mucositis at some stage, while 15%–20% develop peri‐implantitis [5, 6]. By definition, PIDs arise as a complication of a therapeutic intervention. Once established, they exert a non‐negligible impact: they may compromise the functional and esthetic outcomes of implant therapy, requiring additional interventions to manage biological complications; and, in more advanced stages, they may ultimately lead to implant loss, signifying treatment failure. The management of PIDs is particularly challenging, as outcomes remain unpredictable, and recurrence is common. In this context, preventive strategies should be systematically integrated across all phases of implant‐supported rehabilitation, from pre‐surgical assessment to long‐term SPIC, with the goal of minimizing PIDs incidence by addressing the multiple modifiable risk factors and indicators (e.g., inadequate plaque control, smoking, poorly controlled diabetes) the patient may be exposed to [7].

However, specific considerations apply to the prevention of PIDs, as these conditions exclusively affect individuals undergoing implant‐supported rehabilitation for the replacement of missing teeth. Therefore, primordial prevention strategies, which are typically directed toward the general population, should instead be targeted to patients awaiting dental implant placement.

Prevention of PIDs must therefore begin with the careful selection and preparation of patients for implant therapy, ensuring conditions that favor stable osseointegration and long‐term function. In other words, patients should be “prehabilitated” before they are rehabilitated with an implant‐supported prosthesis.

Prehabilitation is not a novel concept in medicine. In general surgery, it refers to a multimodal strategy aimed at controlling modifiable risk factors and optimizing patients' physical, nutritional, and psychological conditions prior to elective surgery in order to improve postoperative recovery and long‐term outcomes [8]. Prehabilitation protocols are increasingly being implemented across several surgical fields and are now recognized as a proactive, cost‐effective strategy to reduce postoperative complications and accelerate recovery, particularly in frail or high‐risk patients. This also translates into lower overall treatment costs and greater patient satisfaction and involvement in their own care [9]. Prehabilitation protocols are implemented in the preoperative period and generally last 2 to 6 weeks to allow meaningful improvements in functional capacity, nutritional status, and comorbidity control prior to surgery.

Applied to implant dentistry, primordial prevention and prehabilitation are complementary and mutually reinforcing concepts. Primordial prevention is applied at the population level, targeting individuals likely to require implant therapy at some point. The goal is to minimize exposure to risk factors and promote good oral health, which is a prerequisite for successful dental implant placement and long‐term peri‐implant health [1, 10]. Indeed, primordial prevention in implant dentistry aligns with the broader goal of preventing tooth loss; effective management of periodontal diseases and caries in the general population will reduce the need for dental implants and thereby the exposure to PIDs risk. On the other hand, prehabilitation is applied at the patient level, functioning as the operational tool to implement primordial prevention of PIDs. Its purpose is to optimize treatment conditions and enhance outcomes because implants will be placed in “ideal conditions,” whenever possible.

Thus, prehabilitation protocols should be structured for patients awaiting dental implant(s), should be tailored on individual patient risk factors, and should include multimodal interventions at both the patient and implant site levels (Figure 1).

The following checklist of procedures may be suggested:

- *Patient information* on dental implant‐supported rehabilitation, covering the surgical process, recovery expectations, potential mechanical and biological complications, and the critical role of SPIC. Candidates for dental implants must recognize PID risk factors, and clearly understand that SPIC represents the most effective strategy to maintain peri‐implant health over time.
- *Oral hygiene optimization* tailored to the patient's individual needs and adapted to the planned rehabilitation.
- *Achievement of optimal oral health*, including comprehensive treatment of oral diseases, such as periodontitis.
- *Lifestyle modifications*, including smoking cessation, improved nutrition, good sleep hygiene, and increased physical activity according to individual's risk profiles.
- *Management of comorbidity*, whenever present, such as diabetes through glycemic control (HbA1c < 7%). Collaboration with physicians, general practitioners, or specialists may be required.
- *Prevention and management of oral parafunctions* (e.g., bruxism) *and psychosocial stress control*. Although evidence is limited, wake‐time clenching, sleep‐related tooth grinding and oral parafunctions, often related to high levels of psychosocial stress and poor coping, may increase the risk of dental implant biological and mechanical complications.
- *Dental implant‐site optimization* to facilitate prosthetically‐driven implant placement and prosthetic design. This may include hard‐ and soft‐tissue augmentation when deficiencies are present (e.g., < 2 mm of peri‐implant keratinized tissue width or peri‐implant bone thickness) [11, 12] as well as adjustments in surgical protocols.

To individualize the prehabilitation protocol, dental implant risk assessment tools or scoring systems [13] can be applied to identify high‐risk patients preoperatively and adapt the required interventions accordingly, also establishing the intensity and duration of the prehabilitation. Ideally, on the day of implant placement, the patient receiving implant surgery should be in the optimal condition to receive it, meaning free of risk factors that could complicate surgery and increase the risk of postoperative complications. Thus, since the implant is the first risk factor for the development of PIDs, whenever the patient's risk profile cannot be adequately controlled or modified to avert risk factors prior to dental implant placement, postponing surgery or considering alternative treatment options (e.g., tooth‐supported prosthesis, conventional dentures, or tooth retention strategies) should be evaluated as possible preventive strategies. In other words, clinicians should consider modifying the treatment plan whenever the risk of PIDs is not acceptably managed preoperatively. If this is not possible, patients should be informed of the risk, and additional preventive measures should be applied after implant placement (e.g., shorter intervals for SPIC).

It is also evident that the proposed list of prehabilitation targets must be regularly updated as knowledge in the field advances. To date, the literature on the effectiveness of primordial prevention for PIDs is lacking [2]. However, it is estimated that patients attending SPIC regularly present with significantly reduced risk of PIDs (Odds ratio, OR: 0.42). Similarly, diabetic patients with good glycemic control show a lower risk of peri‐implantitis (OR: 0.16), while an adequate amount of peri‐implant keratinized mucosa is associated with lower peri‐implant inflammation and lower marginal bone level changes [2].

Thus, by systematically implementing primordial prevention and prehabilitation protocols, both short‐ and long‐term outcomes of implant‐supported rehabilitations may be significantly improved, as implants will be placed and loaded under optimal conditions to ensure long‐lasting functional and esthetic success. Large‐scale prospective and retrospective studies are needed to assess the impact of primordial prevention strategies on short‐ and long‐term outcomes in implant dentistry. However, while further clinical evidence is awaited, it is already evident that primordial prevention and prehabilitation effectiveness rely heavily on the patient's active and sustained engagement in risk factor control. Patient involvement is therefore indispensable to achieving and maintaining peri‐implant health, enhancing treatment satisfaction, and ultimately improving oral health–related quality of life.

## Conflicts of Interest

The authors declare no conflicts of interest.

## Data Availability

The authors have nothing to report.

## References

[1] D. Herrera , T. Berglundh , F. Schwarz , et al., “Prevention and Treatment of Peri‐Implant Diseases‐The EFP S3 Level Clinical Practice Guideline,” Journal of Clinical Periodontology 50, no. Suppl 26 (2023): 4–76. 10.1111/jcpe.13823 37271498

[2] M. C. Carra , N. Blanc‐Sylvestre , A. Courtet , and P. Bouchard , “Primordial and Primary Prevention of Peri‐Implant Diseases: A Systematic Review and Meta‐Analysis,” Journal of Clinical Periodontology 50, no. Suppl 26 (2023): 77–112. 36807599 10.1111/jcpe.13790

[3] G. G. Nascimento , S. Alves‐Costa , and M. Romandini , “Burden of Severe Periodontitis and Edentulism in 2021, With Projections up to 2050: The Global Burden of Disease 2021 Study,” Journal of Periodontal Research 59 (2024): 823–867. 39192495 10.1111/jre.13337

[4] S. Alves‐Costa , M. Romandini , and G. G. Nascimento , “Lip and Oral Cancer, Caries and Other Oral Conditions: Estimates From the 2021 Global Burden of Disease Study and Projections up to 2050,” Journal of Periodontal Research 60 (2025): 544–558. 40530949 10.1111/jre.13421 PMC12312818

[5] G. Baima , F. Romano , S. Chuachamsai , et al., “Prevalence and Risk Indicators of Peri‐Implant Diseases and Buccal Soft‐Tissue Dehiscence: A Cross‐Sectional Study From a University‐Based Cohort,” Journal of Periodontal Research (2025): 70025, 10.1111/jre.70025. PMC13140783 40771021

[6] M. Romandini , C. Lima , D. Banaco , R. Azevedo , and M. Sanz , “Incidence and Risk Factors of Peri‐Implantitis Over Time—A Prospective Cohort Study,” Journal of Periodontal Research (2025): 13367, 10.1111/jre.13367. PMC12881879 39803810

[7] J. H. Fu and H. L. Wang , “Breaking the Wave of Peri‐Implantitis,” Periodontology 2000 84 (2020): 145–160. 32844418 10.1111/prd.12335

[8] F. D'Amico , S. Dormio , G. Veronesi , et al., “Home‐Based Prehabilitation: A Systematic Review and Meta‐Analysis of Randomised Trials,” British Journal of Anaesthesia 134 (2025): 1018–1028. 39919985 10.1016/j.bja.2025.01.010 PMC11947603

[9] N. H. Soh , C. R. Z. Yau , X. Z. Low , et al., “Prehabilitation Outcomes in Surgical Oncology Patients Undergoing Major Abdominal Surgery: A Meta‐Analysis of Randomized Control Trials,” Annals of Surgical Oncology 32 (2025): 1236–1247. 39616295 10.1245/s10434-024-16527-8

[10] M. C. Carra , H. Range , P. J. Swerts , K. Tuand , K. Vandamme , and P. Bouchard , “Effectiveness of Implant‐Supported Fixed Partial Denture in Patients With History of Periodontitis: A Systematic Review and Meta‐Analysis,” Journal of Clinical Periodontology 49, no. Suppl 24 (2022): 208–223. 34775625 10.1111/jcpe.13481

[11] D. S. Thoma , N. Naenni , E. Figuero , et al., “Effects of Soft Tissue Augmentation Procedures on Peri‐Implant Health or Disease: A Systematic Review and Meta‐Analysis,” Clinical Oral Implants Research 29, no. Suppl 15 (2018): 32–49. 29498129 10.1111/clr.13114

[12] I. Sanz‐Sanchez , A. Carrillo de Albornoz , E. Figuero , et al., “Effects of Lateral Bone Augmentation Procedures on Peri‐Implant Health or Disease: A Systematic Review and Meta‐Analysis,” Clinical Oral Implants Research 29, no. Suppl 15 (2018): 18–31. 29498126 10.1111/clr.13126

[13] L. J. A. Heitz‐Mayfield , F. Heitz , and N. P. Lang , “Implant Disease Risk Assessment IDRA‐A Tool for Preventing Peri‐Implant Disease,” Clinical Oral Implants Research 31 (2020): 397–403. 32003037 10.1111/clr.13585

